# Identification of uPAR-positive Chemoresistant Cells in Small Cell Lung Cancer

**DOI:** 10.1371/journal.pone.0000243

**Published:** 2007-02-28

**Authors:** Margarita Gutova, Joseph Najbauer, Anna Gevorgyan, Marianne Z. Metz, Yehua Weng, Chu-Chih Shih, Karen S. Aboody

**Affiliations:** 1 Division of Hematology/Hematopoietic Cell Transplantation, City of Hope National Medical Center and Beckman Research Institute, Duarte, California, United States of America; 2 Division of Neurosciences, City of Hope National Medical Center and Beckman Research Institute, Duarte, California, United States of America; Sanofi-Aventis, United States of America

## Abstract

**Background:**

The urokinase plasminogen activator (uPA) and its receptor (uPAR/CD87) are major regulators of extracellular matrix degradation and are involved in cell migration and invasion under physiological and pathological conditions. The uPA/uPAR system has been of great interest in cancer research because it is involved in the development of most invasive cancer phenotypes and is a strong predictor of poor patient survival. However, little is known about the role of uPA/uPAR in small cell lung cancer (SCLC), the most aggressive type of lung cancer. We therefore determined whether uPA and uPAR are involved in generation of drug resistant SCLC cell phenotype.

**Methods and Findings:**

We screened six human SCLC cell lines for surface markers for putative stem and cancer cells. We used fluorescence-activated cell sorting (FACS), fluorescence microscopy and clonogenic assays to demonstrate uPAR expression in a subpopulation of cells derived from primary and metastatic SCLC cell lines. Cytotoxic assays were used to determine the sensitivity of uPAR-positive and uPAR-negative cells to chemotherapeutic agents. The uPAR-positive cells in all SCLC lines demonstrated multi-drug resistance, high clonogenic activity and co-expression of CD44 and MDR1, putative cancer stem cell markers.

**Conclusions:**

These data suggest that uPAR-positive cells may define a functionally important population of cancer cells in SCLC, which are resistant to traditional chemotherapies, and could serve as critical targets for more effective therapeutic interventions in SCLC.

## Introduction

Small cell lung cancer (SCLC) is the most aggressive type of lung cancer and has a uniformly poor prognosis. Metastases develop quickly, primarily to bone marrow and brain, and are usually present at the time of diagnosis. In untreated patients, median survival is two months from the onset of symptoms [1].

In several types of tumors increased levels of urokinase plasminogen activator (uPA) and its receptor uPAR (CD87) strongly correlate with poor prognosis and unfavorable clinical outcome [2], [3], [4], [5], [6]. uPA and uPAR are instrumental in controlling membrane-associated extracellular proteolysis and transmembrane signaling, thus affecting cell migration and invasion under physiological and pathological conditions [2], [7], [8], [9], [10]. uPAR over-expression in malignant cells results from activation of several oncogenic pathways, including MAPK, RTK, ERK2 and FAK [2], [7], [9]. Multiple oncogenic mutations, including p53 in cancer cells lead to uncontrolled expression of uPA/uPAR [11]. Inhibition of uPAR in a mouse model of non-small cell lung cancer and other tumors inhibited tumor growth, invasion, angiogenesis and metastasis [12], [13], [14]. Increased levels of uPAR are correlated with higher mortality in patients with squamous cell and non-small cell lung cancer [15], [16], however little is known about the role of uPA/uPAR expression in SCLC.

A recent study by Alfano *et al* underlines the importance of uPAR signaling in prevention of apoptosis by resistance of cancer cells to anoikis (apoptosis induced by loss of anchorage). uPAR expression promotes cell survival by activating anti-apoptosis factor Bcl-xL transcription through the MEK/ERK- and PI3K/Akt-dependent pathways [17]. Therefore, we hypothesize that uPAR expression may be involved in development of drug-resistant cancer phenotype in SCLC.

We report here the presence of a rare population of uPAR-positive cells in human SCLC cell lines that demonstrate significant drug resistance to traditional chemotherapeutic agents such as 5-fluorouracil (5-FU), cisplatin and etoposide. The uPAR-positive cells expressed stem- and cancer cell markers, including CD44 and MDR1. Identification and targeting of uPAR-positive cells in SCLC may provide valuable insight into biology of human lung cancer and may establish novel critical targets for more effective anticancer therapies.

## Methods

### Immunostaining and Flow Cytometry Analysis

Primary (lung) small cell lung carcinoma (SCLC) cell lines (H1688, H1417, H69AR), bone marrow (BM) metastatic SCLC (H211, H1882) and brain metastatic SCLC (H250) cell lines were obtained from human primary lung and metastatic tissues ( ATCC), grown in RPMI 1640 modified medium (ATCC, N: 30–2001) supplemented with 10% Fetal Bovine Serum (FBS). The BM metastatic cell line (H1882) was cultured in complete HITES medium (D-MEM/F-12, N: 30–2006 supplemented with insulin 5 µg/mL, transferrin 10 µg/mL, sodium selenite 30 nM, hydrocortisone 10 nM, β-estradiol 10 nM, L-glutamine 2 mM, HEPES 10 mM and 5% FBS). Cells were grown for two weeks and were analyzed by flow cytometry using the following antibodies: CD59 (CBL467P), CD109 (CBL585P), CD62E (CBL180F) from Chemicon, CD87 (3936CJ) from American Diagnostica, CXCR4 (FAB170F) from R&D Systems, CD24 (555427), CD90 (555596), CD38 (347680), CD44 (555478), CD45 (555482), CD13 (555394), CD49b (555498), CD29 (555443), CD3 (30104X) from BD Pharmingen, ABCG2/BCRP1 (10400) from Stem Cell Technologies, CD133/2 (clone 293C3) and CD133/1 (clone AC133) from Miltenyi Biotec, CD34 (347660) from Becton Dickinson, CD105 (326–050) from Alexis, MNF116 (F0859), Cyt18 (F7212) from DACO, and CD166 (3FT) from RDI. For FACS analysis each cell line was detached by trypsinization and re-suspended in staining buffer (SB) (HBSS, Irvine Scientific, 9228) supplemented with 2% FBS and 10 mM HEPES at a density of 5×10^6^ cells/ml. Fifty µl (2.5×10^4^ cells) was added to each well of a 96-well v-shaped plate. Antibodies (FITC- or PE-conjugated) were added in concentrations recommended by the manufacturer (20 µl/10^6^ cells). Antibodies to CD133, CD34, CD44, CD87 and MDR1 have been individually titrated. The 96-well plates were placed on ice and cells were stained with antibodies for 30 min in dark. After staining, 150 µl of wash buffer (HBSS, supplemented with 15% FBS and 10 mM HEPES)/well was added and the plates were centrifuged at 500× *g* for 5 min at 4°C. The cell pellets were re-suspended in SB, supplemented with propidium iodide (PI) (1 µg/ml) to exclude nonviable cells, followed by flow cytometric analysis.

### Cell Staining and Sorting

SCLC cell lines (H211, H69AR, H1417) were grown in RPMI 1640 medium and 4×10^6^ cells were collected and then re-suspended in 800 µl of SB, followed by staining with uPAR (CD87)-FITC-conjugated antibody as described above. uPAR-positive and uPAR-negative cells were sorted by FACS, followed by culture in “base” methylcellulose media (Stem Cell Technologies, 04100) for 16 days at 37°C, 5% CO_2_.

### Clonogenic Assay of SCLC Cell Lines

Complete RPMI 1640 was added to MethoCult H4100 medium (40 ml) to achieve a final volume of 100 ml. uPAR-positive and uPAR-negative cells derived from SCLC cell lines (H211, H69AR, H1417) were plated in MethoCult medium in triplicates containing 3×10^3^, 1×10^3^, or 1×10^2^ cells/ml. Cells were cultured at 37°C, 5% CO_2_ in a humidified incubator for 16 days. Colonies were counted using an inverted brightfield microscope at 4× magnification.

### Cytotoxicity Assay

Cells derived from three SCLC lines were immunostained and sorted by flow-cytometry analysis (as described above). Cell lines (H211, H69AR, H1417) were counted and placed into 96-well plates (4×10^3^ cells/well, in triplicates) with final concentrations of drugs 0, 3, 10, 100, 200 µg/ml. After incubation for 72 hr, both viable and dead cells were counted by using Guava ViaCount assay, and only the viable cells were included in data analysis. The Guava ViaCount assay distinguishes between viable and non-viable cell based on the differential permeability of DNA-binding dyes in the ViaCount reagent, and thus fluorescence of the dyes allows quantitative assessment of both viable and non-viable cells in suspension. Alternatively, non-sorted cells were applied to 48-well plates (1×10^4^ cells/well) and treated with cisplatin, etoposide and their combination in concentrations of 0, 3, 10, 100 µg/ml. The seeding densities per unit area (mm^2^) were the same for both the 48- and 96-well plates (density = 125 cells/mm^2^). After 72 hrs of incubation, viable cells were stained with uPAR-FITC antibodies and evaluated by flow cytometry.

### Double-labeling for Flow Cytometry

Cell lines were stained with CD44-PE and MDR1-PE, were washed and then stained with uPAR-FITC (1×10^5^ cells/1 µg of antibody). Iso-type matched PE- or FITC-conjugated antibodies were used as controls. Stained cells were analyzed by flow cytometry.

## Results

### Flow Cytometry Analysis of Human SCLC Cell Lines

**Figure 1:**
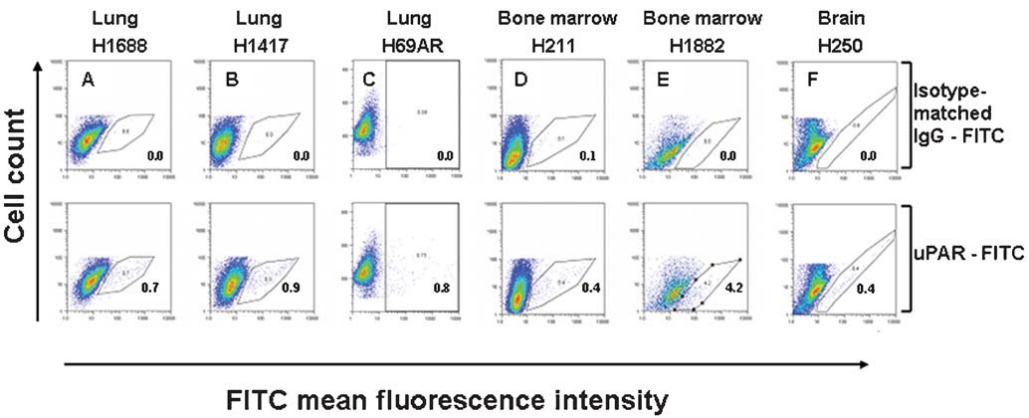
Flow cytometric analysis of uPAR expression in SCLC-derived cell lines. (A, B, C) Lung-derived SCLC cell lines, (D, E) metastatic bone marrow and (F) metastatic brain cell lines. All cells were cultured in RPMI 1640 medium, stained with uPAR-FITC antibody and analyzed by flow cytometry (*lower panels*). Control staining was performed using FITC-conjugated, isotype-matched mouse IgG (*upper panels*). A small population of uPAR-positive cells was detected in all cell lines examined, and is indicated as percent of R1-gated viable cells. Results shown are representative of three independent experiments.

To identify the most invasive SCLC phenotypes, we screened six human SCLC cell lines (H1688, H1417, H69AR derived from primary tumor site in lung, H250 from metastases to brain, and H211, H1882 from metastases to bone marrow) with a panel of antibodies for surface determinants of tumor cells, including uPAR, CD13, CD29, CD44, CXCR4, CD105, CD109, CD166, and for stem cell markers CD34, CD90, CD133, ABCG2/BCRP1. SCLC cell lines displayed heterogeneous phenotypes with regard to tumor surface determinants. All six SCLC cell lines were positive for CD29 (20–99%), CD44 (8–98%), CD105 (3–34%), CD166 (85–98%), and negative for CD90, and CXCR4. uPAR (CD87) was the only cell surface antigen expressed on a small sub-population of cells (1–4%) in each of the six SCLC cell lines, when analyzed by FACS using anti-uPAR-FITC antibody (Figure 1, *lower panels*).

Control samples stained with isotype-matched IgG-FITC were negative for uPAR expression (Figure 1, *upper panels*). The consistent presence of a small uPAR-positive subpopulation of cells in all primary (lung, Figure 1, *lower panels* A, B, C) and metastatic (bone marrow; Figure 1, *lower panels* D, E, and brain; F) SCLC cell lines, in contrast to other markers, which varied in abundance, suggests that uPAR-positive cells may comprise a functionally unique subpopulation of cells.

### Chemoresistance of uPAR-positive Cells

**Figure 2:**
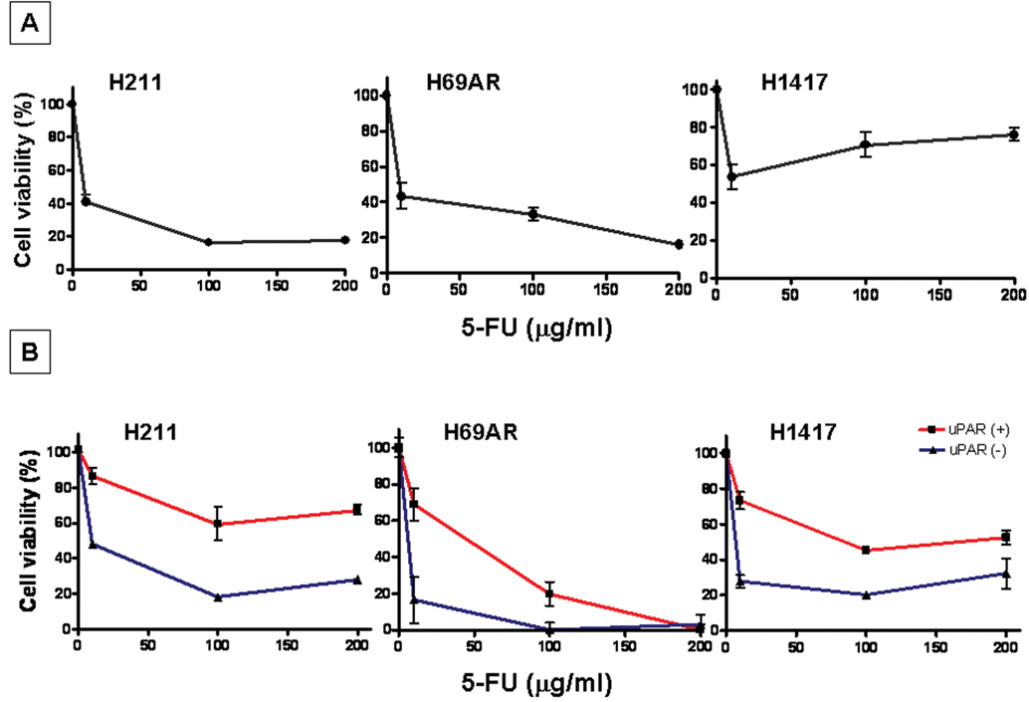
Cytotoxic effect of 5-FU on non-sorted and sorted (uPAR-positive and uPAR-negative populations) derived from SCLC cell lines. (A) 1×10^4^ cells (H211, H69AR, H1417) were placed in wells of a 48-well plate in triplicates and incubated for 72 hr in the presence of varying concentrations of 5-FU. (B) SCLC cell lines were FACS sorted after staining with anti-uPAR antibodies and were plated at the same seeding density (4×10^3^/well of 96-well plate) and treated with 5-FU at 0, 10, 100, 200 µg/ml for 72 hr. Cell survival was evaluated after adding Guava ViaCount reagent and counting viable and dead cells. Only viable cells were included in data analysis, and 100% viability was defined as number of viable cells cultured in absence of 5-FU. Statistical analysis (2-way ANOVA) of uPAR(+) and uPAR(−) data sets revealed significant differences among viability of uPAR(+) and uPAR(−) cells (*P* = 0.0002, 0.0027, 0.0008 for H211, H69AR, H1417 cells, respectively). The data points represent averages±SD of three independent experiments.

We hypothesized that the uPAR-expressing cell population may be resistant to chemotherapeutic agents. We performed cytotoxicity assays on three selected cell lines using non-sorted (bulk) as well as sorted uPAR-positive and uPAR-negative cell populations. Increasing concentrations of 5-fluorouracil (5-FU) (10, 100, 200 µg/ml) were added to these cell cultures and incubated for 72 hrs, followed by counting of both viable and killed cells. Data was normalized to 100%, which signified the number of uPAR-positive and uPAR-negative cells without drug added. A cell-killing effect was detected in all three non-sorted cell lines (H211, H69AR, H1417), where 40–80% of cells were killed by 5-FU (Figure 2A). Importantly, uPAR-positive sorted cells from these three cell lines displayed significantly increased resistance to 5-FU, with only 40–50% of cells being killed in the case of H211 and H1417 (Figure 2B). Although the H69AR cell line also showed differential killing of uPAR-positive and uPAR-negative cells (e.g., 10 µg/ml 5-FU killed 30% of uPAR-positve cells versus 85% of uPAR-negative cells), at high concentration of 5-FU (200 µg/ml), the killing effect for both populations (H69AR) was the same (∼100%). Statistical analysis (2-way ANOVA) of uPAR(+) and uPAR(−) data sets revealed significant differences in survival of uPAR(+) and uPAR(−) cells after treatment with 5-FU (*P* = 0.0002, 0.0027, 0.0008 for H211, H69AR, H1417 cells, respectively) (Figure 2B).

**Figure 3:**
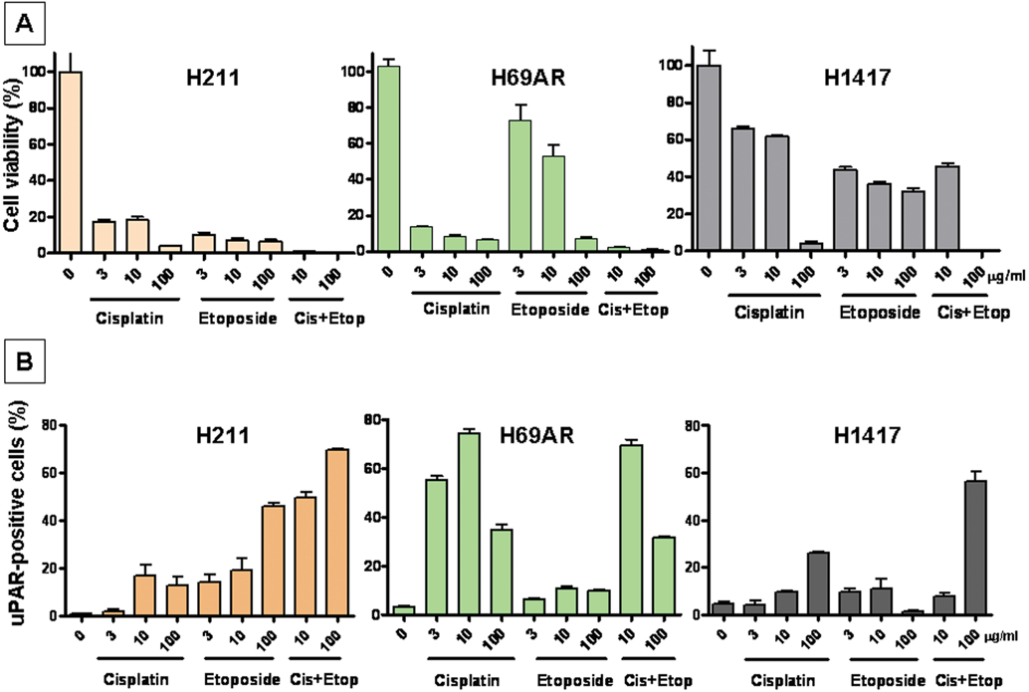
Cytotoxic effect of cisplatin and etoposide on non-sorted cells derived from SCLC cell lines. (A) SCLC cell lines (non-sorted) treated with cisplatin, etoposide at concentrations 0, 3, 10, 100 µg/ml or their combinations (cisplatin and etoposide at final concentrations of 10 µg/ml, 100 µg/ml) for 72 hr. Cell survival was evaluated after addition of Guava ViaCount reagent and counting of both surviving and dead cells using Guava ViaCount software. Data were normalized as 100% viability of cells cultured in absence of drugs. Error bars indicate standard deviation of triplicate cultures (results of three independent experiments). (B) After treatment cisplatin and etoposide, viable adherent cells were detached by trypsin treatment and were stained with anti-uPAR-FITC antibodies and percentage of uPAR-positive cells was determined by FACS analysis. Sample with mouse IgG isotype control antibody was used to set the value of the FACS gate, which was applied to all samples stained with uPAR-FITC.

We similarly investigated the cytotoxic effect of cisplatin and etoposide, drugs currently used for treatment of patients with SCLC. The non-sorted SCLC cell lines (H211, H69AR, H1417) were used in cytotoxic assays with cisplatin and etoposide in *in vitro* studies (3, 10, 100 µg/ml) (Figure 3A). 60–80% of cells were killed by cisplatin and etoposide (used separately or in combination) in non-sorted lines (Figure 3A). Again, the uPAR-positive sorted cells from these three cell lines displayed significantly increased resistance to cisplatin and etoposide, with only 30–50% of cells being killed, compared to 60–80% killing of the uPAR-negative sorted cells (data not shown). These data suggest that the uPAR-positive cell subpopulation in SCLC may be responsible for chemoresistance to 5-FU and other traditional chemotherapeutic drugs such as cisplatin and etoposide.

In order to confirm that uPAR expression confers tumor resistance to cisplatin and etoposide, we performed cytotoxicity assays on the same non-sorted SCLC cell lines and looked for enrichment of uPAR-positive cells in the surviving cell population. Indeed, we detected a significant enrichment of uPAR-positive cells (40–70%) among surviving cells, compared to only 1–5% of uPAR-positive cells in control cultures grown in the absence of these drugs (Figure 3B). These data support the hypothesis that uPAR expression confers chemoresistance in SCLC, and that the uPAR-positive cells should be considered as a novel target for more effective therapies.

### Clonogenic Activity of uPAR-positive Cells

**Figure 4:**
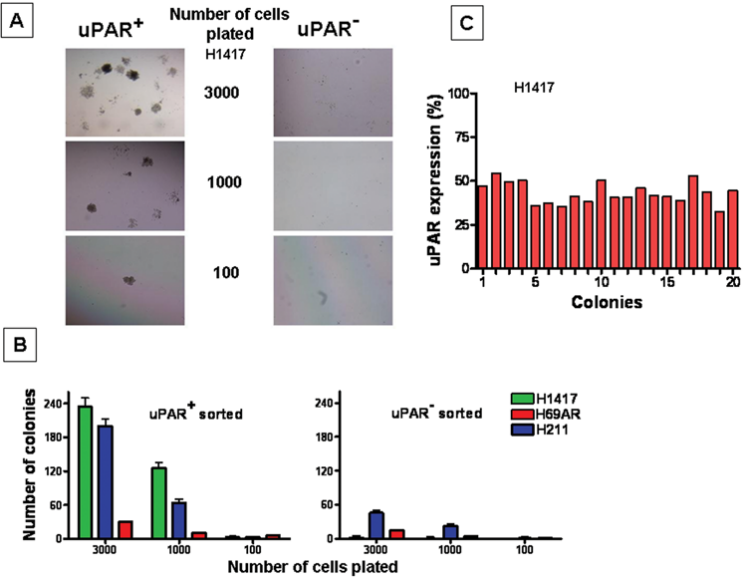
Colony-forming activity of uPAR-positive and uPAR-negative cells derived from SCLC cell lines. (A) H1417- derived, uPAR-positive sorted cells formed multiple colonies in methylcellulose media, while uPAR-negative cells from the same sorts displayed little or no clonogenic activity. (B) Graphical representation of colony-forming ability of uPAR-positive and uPAR-negative cells at different plating densities 3000, 1000, 100 cells/6-well plate (H1417, H69AR, H211). (C) Distribution of uPAR-positive cells in the colonies derived from sorted uPAR-positive cells grown in methylcellulose media. A total of 20 cell colonies from the H1417 cell line were analyzed.

To show that uPAR-positive cells possess high clonogenic and self-renewal potential, we sorted primary lung (H1417, H69AR), metastatic bone marrow (H211) cell lines by FACS using anti-uPAR-FITC-conjugated monoclonal antibodies. After sorting (97% purity), uPAR-positive and uPAR-negative cells were plated in methylcellulose media at densities of 3000, 1000, 100 cells/well (6-well plate). uPAR-positive cells from these primary and metastatic SCLC lines formed multiple distinct colonies (Figure 4A). We estimated that approximately ∼10% of the uPAR-positive cells (lung, H1417; bone marrow, H211) formed colonies (Figure 4B). Conversely, the uPAR-negative cells from the primary lung (H1417) cells did not form colonies, and from metastatic bone marrow (H211) and lung (H69AR) formed only few colonies (Figure 4B). After uPAR-positive cells were allowed to form colonies for 16 days in methylcellulose culture, 20 colonies were isolated and analyzed for uPAR expression by flow cytometry. We found both uPAR-positive and uPAR-negative cells in each colony analyzed (H1417), with 30–50% of cells displaying uPAR positivity within each colony, following 18 days of incubation (Figure 4C). This suggests that uPAR-positive cells can give rise to both uPAR-positive and uPAR-negative cells. uPAR expression was also analyzed in the colonies derived from H211 and H69AR cell lines. In all colonies derived from sorted uPAR-positive cells we detected both uPAR-positive and uPAR-negative cells (∼50% each after 16 days in methylcellulose culture) (data not shown). Analysis of colonies derived from uPAR-negative cells (e.g., H211 cell line) revealed a small proportion of uPAR-positive cells in those colonies (<1%). The reason for this might be possible contamination during sorting. Alternatively, uPAR-negative cancer cells may acquire uPAR expression under certain culture conditions. Several uPAR-positive colonies have been picked and grown further for 2 weeks in RPMI culture medium, followed by FACS sorting and clonogenic assay. Interestingly, we detected a similar proportion of uPAR-positive and uPAR-negative cells (1–5% and 95–99%, respectively) in these cultures, suggesting that uPAR-positive colonies can recapitulate the parental cell phenotype. In cultures derived from uPAR-negative colonies on the other hand, we detected lower levels (0.1–0.8%) of uPAR-positive cells, and poor overall growth (data not shown).

### Co-expression of uPAR with CD44 and MDR1

**Figure 5:**
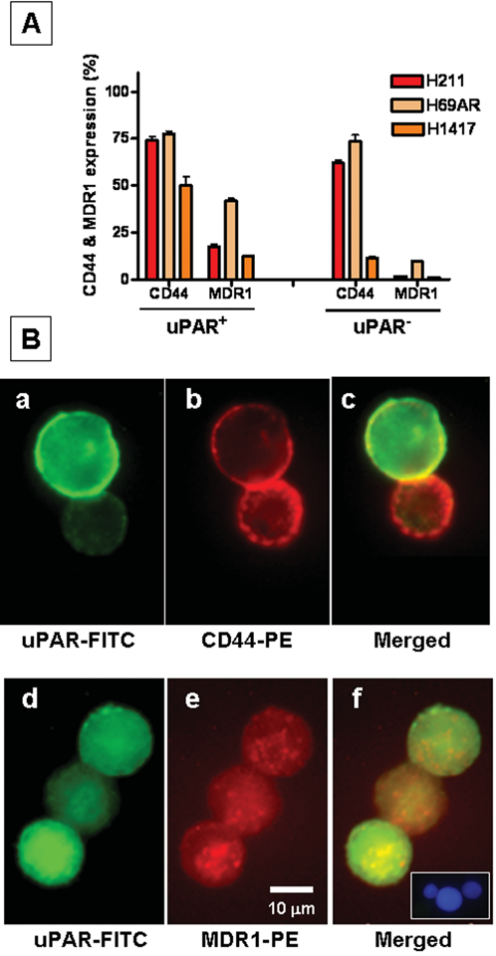
Expression of CD44 and MDR1 on uPAR-positive and uPAR-negative cells. (A) FACS analysis of, H211, H69AR and H1417 SCLC cell lines double-labeled with uPAR-FITC and CD44-PE, MDR1-PE. The percentages of cells expressing CD44 and MDR1 were calculated separately for uPAR-positive and uPAR-negative cells. (B) Fluorescent microscopic analysis of double-labeled and FACS-sorted cells. Examples of uPAR-FITC/CD44-PE double-labeling (a,b,c) and uPAR-FITC/MDR1-PE double-labeling (d,e,f). (Bf-inset) H1417 cell line stained with mouse IgG isotype control-PE (red), isotype control-FITC (green) and DAPI (blue).

We further hypothesized that if uPAR-positive cells represent drug-resistant and clonogenic population in SCLC, they may express CD44 and MDR1 (ABCB1) genes, which are involved in development of cancer stem cell phenotype and multi-drug resistance. To further characterize the uPAR-positive population, we performed double-labeling experiments with uPAR-FITC, and CD44-PE as well as MDR1-PE on H211, H69AR, H1417 cell lines. uPAR-positive cells derived from SCLC cell lines (H211, H69AR, H1417) expressed CD44 on 50–80%, whereas MDR1 on 10–40% of cells (Figure 5A). Expression of the above markers was also confirmed using fluorescent microscopy (Figure 5B). The uPAR-negative cells also expressed CD44 (∼70% for H211 and H69AR cells; ∼10% for H1417) and MDR1 (1–10% for all cell lines) (Fig. 5A). These data suggest an enrichment of CD44 expression on H1417 cells, whereas MDR-1 showed increased expression on uPAR-positive cells derived from all three cell lines compared to uPAR-negative cells (Fig. 5A).

## Discussion

The main finding of this study is identification of a rare subpopulation of uPAR-positive cells in SCLC cell lines derived from lung and metastasis to bone marrow and brain. It is well documented that uPAR over-expression in various malignant tumors is strongly correlated with most invasive cancer phenotypes and poor prognosis [2], [4], [15], [18].

Several studies suggest the presence of a rare population of cells in solid tumors and leukemia that possess the capability to regenerate and propagate the tumor [19], [20], [21], [22], [23]. These cells may also be responsible for maintaining the tumor's malignant potential and serve as the underlying cause of tumor recurrence [24], [25]. Current treatment strategies may fail to target the drug-resistant subpopulation, and could explain the initial therapeutic response of the majority of tumor cells, which is followed by a later recurrence.

We found that uPAR-positive cells were more resistant to treatment with the cytotoxic agent 5-FU, while uPAR-negative cells were killed much more efficiently. The cytotoxic effect of 5-FU has been ascribed to misincorporation of fluoronucleotides into RNA and DNA and to inhibition of the nucleotide synthetic enzyme thymidylate synthase, which mainly targets the fast dividing cells [26]. We also investigated cisplatin and etoposide, chemotherapeutic drugs currently used in treatment of SCLC patients. Importantly, culturing of SCLC cells in the presence of cisplatin and etoposide resulted in selective killing of uPAR-negative cells with concomitant enrichment of the uPAR-positive cell population.

uPAR-positive cells isolated from three SCLC lines were able to proliferate and form multiple colonies in methylcellulose media, while uPAR-negative cells displayed little or no clonogenic potential. We also showed co-expression of uPAR with CD44 and MDR1, which may explain the association between advanced malignancy and drug resistance. Several studies have investigated individually the role of uPAR, CD44 and MDR1 in various malignancies [5], [13], [14], [17], [19], 24,27,28,29,30,31,32, however, to our knowledge this is the first study that provides evidence for expression of CD44 and MDR1 on uPAR-positive cells in SCLC. We also detected CD44 and MDR1 expression on uPAR-negative cells, the functional implications of which remains to be determined.

ATP-binding cassette (ABC) drug transporters have been shown to protect cancer stem cells from chemotherapeutic agents 33. A major transporter of the ABC family is P-glycoprotein, the product of the MDR1 gene, which is produced by hematopoetic stem cells (HSC) 32. The MDR1 gene becomes down-regulated on HSC upon cell differentiation 32. P-glycoprotein and CD44 have been characterized and are known to be determinants of multi-drug resistance on cancer cells, which is mediated by physical and genetic interactions between CD44 and MDR1 30. Activation of CD44 occurs through heterodimerization of CD44 with growth factor receptors (e.g., EGFR, FGFR, HGFR, VEGFR, TGF-βR), which leads to activation of MAP kinase and PI3K-AKT signaling pathways 34. CD44 stimulation by its ligand hyaluronan up-regulates the expression of uPA and uPAR mRNA, through activation of MAPK-Ras pathway, while PI3K activation stimulates MDR1 expression and function 34. PI3K also acts as a positive feedback loop to stimulate hyaluronan production, which activates CD44 35,36. The CD44-MAPK-PI3K signaling leads to uncontrolled expression of uPA/uPAR and MDR1, which promotes invasive and multi-drug resistant cancer cell phenotype. In addition to the CD44-MAPK-PI3K signaling, uPAR over-expression can induce cell survival by activating the anti-apoptosis factor Bcl-xL transcription 17.

Future studies in animal models will address whether the uPAR-positive cells are responsible for primary tumor growth and formation of distant metastases in SCLC. In summary, we have identified the uPAR-positive subpopulation of SCLC cells that possess high multi-drug resistance and clonogenic activity, while the uPAR-negative cells are sensitive to chemotherapeutic drugs and display little or no clonogenic activity. We also provide evidence of association of uPAR, CD44 and MDR1 expression on SCLC cells. Further investigation is warranted to determine if targeting of this cell population will be critical for therapeutic success.
